# Impact of long-range transport on black carbon source contribution and optical aerosol properties in two urban environments

**DOI:** 10.1016/j.heliyon.2023.e19652

**Published:** 2023-09-05

**Authors:** Agnė Minderytė, Emeka A. Ugboma, Fátima Francisca Mirza Montoro, Iwona S. Stachlewska, Steigvilė Byčenkienė

**Affiliations:** a SRI Center for Physical Sciences and Technology (FTMC), 10257 Vilnius, Lithuania; b Faculty of Physics, University of Warsaw (UW), 02-093 Warsaw, Poland

## Abstract

Urban areas, as major sources of aerosol black carbon emissions, contribute to increased pollution levels in surrounding regions by air mass long-range transport, which should be taken into account in implementation of emission-reduction strategies. Properties of light-absorbing aerosol particles and a novel approach to assess the impact of long-range transport on black carbon (BC) pollution in two under-investigated urban environments: Warsaw (Poland, Central Europe) and Vilnius (Lithuania, North-Eastern Europe) are presented. During the warm season of May–August 2022, BC mass concentration and aerosol optical properties: the scattering Ångström exponent (SAE), absorption Ångström exponent (AAE), and single scattering albedo (SSA) were investigated. Generally, the mean BC mass concentration was higher at the more polluted site in Warsaw (1.07 μg/m^3^) than in Vilnius (0.77 μg/m^3^). The BC source apportionment to biomass burning (BC_BB_) and fossil fuel combustion (BC_FF_) showed similar contributions for both sites with BC_BB_ (13–19%) being significantly lower than BCFF (81–87%). A uniform flow of air masses transporting aerosol particles over long distances to both sites was observed for 42% of the days. It affected BC mass concentration as follows: BC decrease was found similar at both sites (42% in Warsaw, 50% in Vilnius) but increase was twice higher in Vilnius (64%) than in Warsaw (30%). Despite variations in BC mass concentration, both sites exhibited a comparable abundance (90%) of submicron (SAE<1.3), BC-dominated (AAE<1.5) particles. The mean SSA was very low (0.69 ± 0.1 in Warsaw, 0.72 ± 0.1 in Vilnius), which indicates a very strong contribution of light-absorbing aerosol particles in both environments. The local episodes of biomass burning due to celebrations of May Days on 1st – 3rd May in Warsaw and Midsummer on 24th June in Vilnius showed similar aerosol properties in both cities (1.5<AAE<1.7, 1.7<SAE<2.2) but were highly different than any other during the entire campaign.

## 1 Introduction

Black carbon (BC) is an atmospheric pollutant of significant local, regional, and global concern in terms of air quality, human health, and climate change [[1], [2], [3], [4], [5], [6]]. The influence of BC greatly depends on its physical properties (e.g., size distribution and morphology) and chemical properties (e.g., mixing state) [4,7].

BC is formed during incomplete combustion from anthropogenic sources – such as fossil fuels (diesel, petroleum, gas, and coal) and biomass burning (e.g., wood or peat pellets, agricultural field fires) – as well as from natural sources (e.g., wildfires), and it accounts for a significant proportion of ambient fine particulate matter (PM_2.5_) [8]. BC can be transported not only locally but across large, even intercontinental distances [9,10]. According to the World Health Organisation (WHO), short-term population exposure to 10 μg/m^3^ of BC can raise mortality from cardiovascular diseases by 0.9% and from other causes by 0.7%, while long-term exposure to 1.0 μg/m^3^ increases the risk of death by 6.0% [8,[11], [12], [13], [14]].

Fossil fuel (FF) combustion and biomass burning (BB) are significant sources of BC in urban areas [14]. Despite the fact that biomass burning is considered to be carbon-neutral energy since it releases the CO_2_ previously absorbed from the atmosphere by plants through photosynthesis, it should not be considered neutral in terms of particulate matter (PM) or BC pollution [15]. Countries around the world have not specified air quality standards for BC concentrations, although monitoring of BC is one of the most important observables at urban sites according to the recent proposal for a European Commission *Directive on ambient air quality and cleaner air for Europe* [16].

In Europe, more 1-hr resolved measurements are reported to the European Monitoring and Evaluation Programme (EMEP) network for rural environments than for urban environments [15,[17], [18], [19], [20], [21], [22], [23]]. The proportion of biomass-related BC sources varies greatly across different areas. According to data from the EU emission inventory report for 2021 under the UNECE Convention on Long-range Transboundary Air Pollution (LRTAP), BC emissions in Poland and Lithuania were primarily from residential and institutional sources (7.53 Gg (45%); 1.08 Gg (57%)) and transport (5.00 Gg (30%); 0.49 Gg (26%)) [23]. Long-term BC trends have been documented for both background (e.g., Preila, Lithuania; [24]) and urban industrial regions (e.g., Zabrze, Poland; [25]).

Aerosol studies over Lithuania have so far been mostly focused on examining the physico-chemical properties, sources, and types of aerosol and their climate implications in the urban background environment [[26], [27], [28], [29], [30], [31]]. Studies on BC source apportionment have shown that in Vilnius, 51–65% of BC mass concentration originates due to traffic emissions and fossil fuel combustion [32,33]. Although apportionment methods have limitations due to the assumption of the absorption Ångström Exponent (AAE), which is influenced by factors such as fuel composition, combustion efficiency, particle size and mixing state with other aerosol constituents [34,35], this method is successfully and widely used for long-term assessment of BC source apportionment [[36], [37], [38]]. More recently, considerable focus has been placed on studying human exposure in urban environments [32,39]. In Poland, however, there is a great need for BC studies in urban environments because there are currently no systematic long-term studies on light-absorbing aerosol optical properties and source apportionment of the light-absorbing aerosols. At the site normally characterized by rural background conditions located at Gwoździanka Hill, South Eastern Poland, BC was identified only during short-term smog events [39]. BC observations have also been reported as long-term measurements in Kraków [40], Zabrze [41], and a short-term study in Warsaw [42], where mean concentrations up to 3.9 μg/m^3^ and maximum concentrations up to 49.9 μg/m^3^ were recorded.

In situ measurements of the optical and physical properties of aerosols have never been conducted in Poland and Lithuania using the present instrument setups (i.e., aethalometer and nephelometer) and the methodological approach. Given the importance of aerosol characteristics to climate and human health, we sought to fill a knowledge gap. The objectives of our study, therefore, are as follows: a) to seek a better understanding of the variation in the source apportionment obtained through systematic in-situ measurements in these urban environments during the warm season; b) to analyse the characteristics of light-absorbing aerosol particles (i.e., BC mass concentration, scattering Ångström Exponent (SAE), absorption Ångström exponent, and single scattering albedo (SSA)) in Vilnius and Warsaw; and c) to take advantage of the two relatively nearby sites and to evaluate the potential impact of long-range air mass transport on BC mass concentration and BC source contributions.

## 2 Experimental sites description and instrumentation

### 2.1 Geographical locations

Observations were conducted from May 1, 2022 to August 18, 2022 in two capital cities in Central Europe (Warsaw, Poland) and North-eastern Europe (Vilnius, Lithuania) . Under the Köppen-Geiger Classification, Warsaw and Vilnius share a similar climate type: humid continental climate (classification index: Cfb for Warsaw and Dfb for Vilnius). However, Warsaw exhibits mildly warmer climate compared to Vilnius. The mean monthly temperatures for the investigated period from May to August range from 15 °C to 20 °C in Warsaw [43] and from 13 °C to 19 °C in Vilnius [44]. In both cities July is the hottest month of the year and sees the highest number of rainy days (13 days in Warsaw and 10 days in Vilnius). Warsaw is Poland's largest city, with a population of 1.9 million residents living in municipality area of about 520 km^2^ (3600 residents/km^2^) (in 2022) [45]. Note that Warsaw is surrounded by many satellite cities (1 million residents) bordering directly with the municipality area referred to together as Warsaw metropolitan area (4200 km^2^). Vilnius, in turn, is Lithuania's largest city, but in contrast to Warsaw, it is significantly less densely populated (1460 residents/km^2^), with 0.6 million residents over only a slightly smaller area of about 400 km^2^ (in 2022) [46].

The Warsaw measurement site of the Faculty of Physics at the University of Warsaw (52°13′ N, 20°54′ E; 112 a.s.l.) is located about 3 km southwest of the city centre, surrounded by commercial and residential buildings, green municipal parks, and a University Campus Park, without obvious effects from industry (thus considered an urban site). The main pollution sources at the Warsaw site are residential and transport activities (more details in Ref. [47]). The Vilnius measurement site, in turn, is located in the low-energy building of the SRI Centre for Physical Sciences and Technology (54°43′ N, 25°19’ E; 131 a.s.l.) about 6 km northeast of the city centre, surrounded mainly by residential and science campus buildings and forested areas, thus considered an urban background site. The main pollution sources at this site are also residential and transport activities (more details in Ref. [38]).

### 2.2 Instrumentation

The BC mass concentration was measured simultaneously at both sites using the same type of aethalometer (Magee Scientific, model AE33), which measured the optical attenuation of aerosol particles sampled on the filter continuously, in real-time [48]. At both sites the sampling resolution for this study was set to 1 min and the flow rate to 4.9 l/min. BC is a principal light absorber at 880 nm and other aerosol components (mineral or carbonaceous) exhibit significantly lower absorption at this wavelength [48]. The BC mass concentration was calculated from light attenuation at 880 nm and the wavelength-specific attenuation cross-section of 7.77 m^2^/g (set by the manufacturer) [49]. The measurement bias caused by filter effects (i.e. the correction for scattering of the filter fibres, scattering of the aerosol particles on the filter, and filter loading effect) are done automatically by the instrument. The lower detection limit of the instruments is 0.1 μg/m^3^ and the upper detection limit is 100 μg/m^3^ [50].

The scattering properties of aerosols were measured with different nephelometers at the same sampling frequency of 30 s. In Vilnius, an integrating nephelometer (TSI, model 3563; [51]) measured aerosol light scattering coefficients (*b*_*scat*_) at 450, 550, and 700 nm for total light scattering angles 7°–170° (selectable angles for backscatter only between 7°–90°). The lower detectable limit is 0.5 Mm^−1^. The sample flow rate is approximately 23 l/min. In Warsaw, a polar nephelometer (Ecotech, model Aurora 4000; [52]) measured *b*_*scat*_ at 450, 525, and 635 nm for total light scattering angles 9°–170° (selectable 17 angles between 10°–90°, 18th angle at 0° which is the standard). The detection limit is 0.3 Mm^−1^ over 60 s integration. The sample flow rate is approximately 5 l/min.

At both sites, we followed the recommendations of ACTRIS (The Aerosol, Clouds and Trace Gases Research Infrastructure) regarding the instrument requirements and quality assurance [53]. We perform regular monthly maintenance for each instrument. The cleaning and calibration of nephelometers with CO_2_ gas is performed every 6 months. The measurements were performed using new aethalometers that have been calibrated by the manufacturer less than 6 months prior to the beginning of our campaign. As for the data curation and treatment, we performed data quality assessment by removing values that are negative or below the detection limit of each instrument.

## 3 Methodology

### 3.1 BCE source apportionment

The BC source apportionment method categorises sources into biomass burning and fossil fuel combustion [35] based on aethalometer measurements and a pre-defined source-specific AAE. Differences in aerosol particle light absorption throughout 370–950 nm can be calculated [[54], [55]]. The AAE is a carbonaceous aerosol particle characteristic that describes how particle light absorption depends on the wavelength, as in Eq [1].

(1) $$b_{abs}\left(\lambda \right)=b_{0}\lambda^{-AAE}$$

where *λ* indicates wavelength, *b*_abs_ the aerosol absorption coefficient, and *b*_0_ is a wavelength-independent constant.

The absorption coefficients of BC attributable to each source (FF or BB) were determined using Eqs [2,3,35].:

(2) $$b_{abs\;FF}=\frac{b_{abs}\left(\lambda_{1}\right)-b_{abs}\left(\lambda_{1}\right)\times {\left(\frac{\lambda_{1}}{\lambda_{2}}\right)}^{-AAE_{BB}}}{{\left(\frac{\lambda_{1}}{\lambda_{2}}\right)}^{-AAE_{FF}}-{\left(\frac{\lambda_{1}}{\lambda_{2}}\right)}^{-AAE_{BB}}}$$

(3) $$b_{abs}\left(\lambda_{1}\right)=b_{abs\;FF}\left(\lambda_{1}\right)+b_{abs\;BB}\left(\lambda_{1}\right)$$

where *b*_*abs*_*(λ*_*1*_*)* denotes total absorption at wavelength *λ*_*1*_, *b*_*abs BB*_ the absorption coefficient of biomass-burning-related BC, and *b*_*abs FF*_ the absorption coefficient for BC from fossil fuel combustion at a wavelength *λ*_*1*_. For our study, *λ*_*1*_ was selected to be 470 nm, while *λ*_*2*_ was 950 nm based on the most commonly used wavelength pair in the literature [54, 55]. The source-specific AAE values differ across regions based on commonly used fuels, fuel origin, and climate conditions [47,49]. Minderytė et al. [32] has previously determined the most suitable combination of AAE values of 0.9 and 2.2 (AAE_FF_ and AAE_BB_, respectively) for BC source apportionment in Vilnius urban background environment. Thus, in this study, the exact same combination was used. For the more polluted urban site in Warsaw, the AAE_FF_ and AAE_BB_ values were 1.0 and 2.0, respectively. The selected values have been widely used in previous studies [49,56,57] and are also chosen as default AAE value combination by the manufacturer of the aethalometer [49].

### 3.2 Aerosol optical properties

The SAE values were calculated for nephelometer data at 450 and 525 nm in Warsaw and 450 and 550 nm in Vilnius, using Eq [4].:

(4) $${\text{SAE}}_{\left(\lambda_{1}/\lambda_{2}\right)}=\frac{\ln\left(\frac{b_{scat}\left(\lambda_{1}\right)}{b_{scat}\left(\lambda_{2}\right)}\right)}{\ln\left(\frac{\lambda_{1}}{\lambda_{2}}\right)}$$

The AAE values were calculated for wavelengths 532 nm and 660 nm using absorption coefficients from aethalometer measurements (Eq. [5]). For this reason, *b*_*abs*_ at 532 nm was evaluated using the method by Zhuang et al. [58] (Eq. [6]):

(5) $${\text{AAE}}_{\left(\lambda_{1}/\lambda_{2}\right)}=\frac{\ln\left(\frac{b_{abs}\left(\lambda_{1}\right)}{b_{abs}\left(\lambda_{2}\right)}\right)}{\ln\left(\frac{\lambda_{1}}{\lambda_{2}}\right)}$$

(6) $$b_{abs}\left(532\;nm\right)=b_{abs}\left(520\;nm\right)\times {\left(\frac{532}{520}\right)}^{-{AAE}_{590/520}}$$

The SSA, describing the relationship between light scattering and absorption, calculated at 525 nm for Warsaw and at 550 nm for Vilnius, was obtained as in Eq [7,59].

(7) $$\text{SSA}=\frac{b_{scat}}{b_{scat}+b_{abs}}$$

where *b*_*scat*_ is the scattering coefficient derived from the nephelometer and *b*_*abs*_ is the absorption coefficient from the aethalometer. The SSA is used in numerous studies to assess aerosol radiative forcing for climate modelling [[60], [61]], which is outside of the scope of our study.

In the present study, an aerosol classification scheme based on thresholds on the SAE plotted versus the AAE, as in the original study by Cappa et al. [62], to investigate the source contribution and chemical composition of aerosol particles. AAE indicates the chemical composition of an aerosol: lower AAE values (1 < AAE <1.5) are BC-dominated, whereas higher AAE values (AAE >1.5) indicate BC mixed externally with brown carbon (BrC) [62], and even greater AAE values (AAE ≥2.5) indicate BrC [63]. Due to the SAE dependence on aerosol particle size, particles can be assigned to size-allocated regions based on dominant SAE values. The SAE is closely related to aerosol particle size, and the value of 1.3 is used as the threshold for discriminating particles: SAE >1.3 indicates submicron (***D***_***a***_<1 μm) particles, and SAE <1.3 indicates supermicron (***D***_***a***_>1 μm) particles [62,64]. Furthermore, according to Cappa et al. [62], submicron particles dominated by BC have SAE >1.8, whereas supermicron particles have SAE <0.2. Based on these thresholds, the data can be separated into four scenarios: “BC dominated” (1<AAE<1.5, AAE < SAE), “Large particle/BC mix” (1<AAE<1.5, AAE>SAE), “Mixed BC, BrC” (1.5<AAE, −1<SAE<1.5), and “Mixed dust/BC/BrC” (1.5<AAE<2, 1.5<SAE<3.5) [62].

### 3.3 Long-range transport of air masses

A new approach is proposed for analysis of backward air mass trajectories to assess impact of long-range transport on local pollution. The Hybrid Single-Particle Lagrangian Integrated Trajectory (HYSPLIT) model [65] was used to calculate 72-hr isentropic backward trajectories (using GDAS1 input) ending at 500 and 1000 m a.s.l. at the locations of the Warsaw and Vilnius sites at 12 UTC for each day over the entire sampling campaign (May–August 2022). Analysis of 72-hr trajectories assured that not only local but also long-range transport aerosol sources were considered. The obtained trajectories were inspected in terms of their uniformity (i.e., overlapping in height and/or horizontality) and assigned to predefined categories. This was done based on thresholds: the direction threshold (defined as the same direction if the trajectories were centred ± 30° tolerance from the end-locations), the horizontal uniformity, here called H-threshold (defined as distance between trajectories equal to 430 km, considered a straight-line distance between Vilnius and Warsaw), and the vertical uniformity, here called V-threshold (defined as a difference in altitude equal a half-height tolerance, i.e. 250 m for the lower trajectories and 500 m for the higher trajectories). In category A, a similar aerosol particle source is possible due to similar or overlapping air mass transport pathways. While air masses arriving at the two sites from different directions and pathways were assigned to category B. Category C, in turn, denotes cases that could not be clearly classified as either “overlapping” or “strictly differing”. All calculated backward trajectories are published in Minderytė et al. (2023).

## 4 Results

### 4.1 Monthly, weekly, and diurnal BC mass concentration variations

**Fig. 1:**
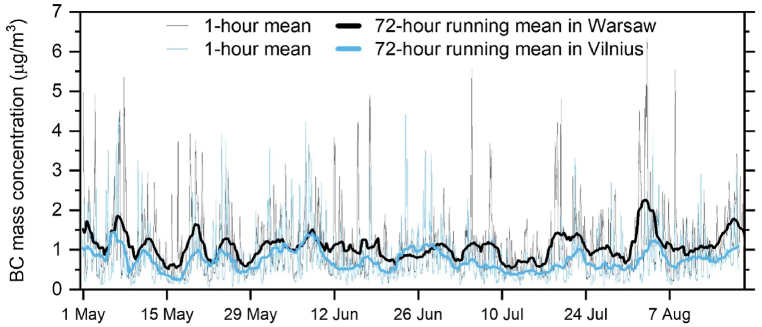
Time series of BC mass concentration in Vilnius (blue) and Warsaw (black) in May–August 2022 for 1-hr mean (thin lines), and 72-hr running mean.

For the warm season of 2022 the measurement series of BC mass concentrations are shown in Fig. 1. The concentrations over Warsaw (black) are higher than those over Vilnius (blue), both for 1-hr mean and 72-hr running mean data. The 72-hr running mean time series allows us to observe longer term BC pollution boosts or declines which are influenced not only by rapid local emissions but also long-range transport. The mean BC mass concentration in Warsaw was 1.07 μg/m^3^ which is 38% higher than in Vilnius (0.77 μg/m^3^). The BC mass concentration during the measurement campaign in Vilnius is comparable to the results obtained in previous study conducted during the warm season in 2020 (0.69 μg/m^3^) [31]. No comparative study had been conducted before in Warsaw, as mentioned earlier, making our study the first one. In both sites, we found no days which could be denoted as high-pollution episodes, here defined as daily mean BC mass concentration above 15 μg/m^3^ (threshold for PM_2.5_ specified in the air quality guidelines of the WHO [66]).

**Fig. 2:**
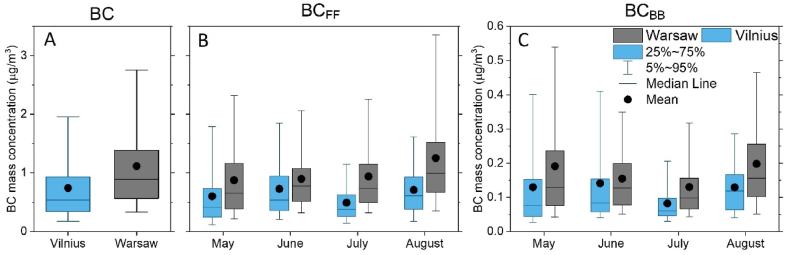
Box and whisker plots of the BC mass concentration in Vilnius (blue) and Warsaw (grey) in May–August 2022. The total BC variability (A), the monthly variability of BC_FF_ (B) and BC_BB_ (C); note the different scales.

The monthly variation of BC mass concentrations and differences in BC source contributions are illustrated in Fig. 2. Not only is the mean BC mass concentration higher in Warsaw, but also the variability in Warsaw is higher (the range within 25th and 75th percentile: from 0.56 to 1.39 μg/m^3^) as compared to Vilnius (from 0.34 to 0.93 μg/m^3^) (Fig. 2(A)). The behaviour is similar in terms of the monthly variability of apportioned BC_FF_ (Fig. 2 (B)) and BC_BB_ (Fig. 2(C)), with BC_FF_ clearly dominating. Month-to-month, BC_FF_ is similar at each site, except for August in Warsaw. In contrast, BC_BB_ shows more vivid changes and similar variability in mass concentration over the two sites (0.13 μg/m^3^ and 0.16 μg/m^3^ in Vilnius and Warsaw, respectively). While in Warsaw, BC_BB_ decreases from May to July and then increases in August, in Vilnius BC_BB_ remains similar for all months, except for a strong reduction in July.

**Fig. 3:**
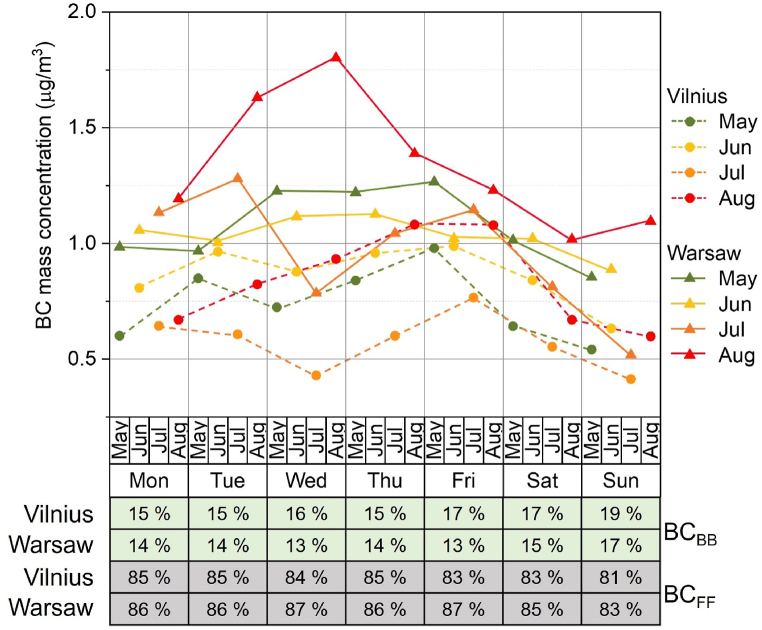
Mean weekly variation in BC mass concentration grouped by month in Vilnius (circles, dashed lines) and Warsaw (triangles, solid lines). The mean daily contributions of BC_BB_ (green) and BC_FF_ (grey) to total BC are given below the chart.

The weekly BC mass concentration is shown in Fig. 3. For Warsaw (solid lines) and Vilnius (dashed lines), the weekly cycles show very similar shapes in July but different shapes in other months. The BC_BB_ contribution is significantly lower (13–19%, highest on weekends), than the dominating BC_FF_ (81–87%, highest on weekdays). This indicates that both contributions may be strongly connected to local activities (weekday traffic, weekend bonfires, and barbeques), although the effect of long-range transport cannot be excluded at this stage.

**Fig. 4:**
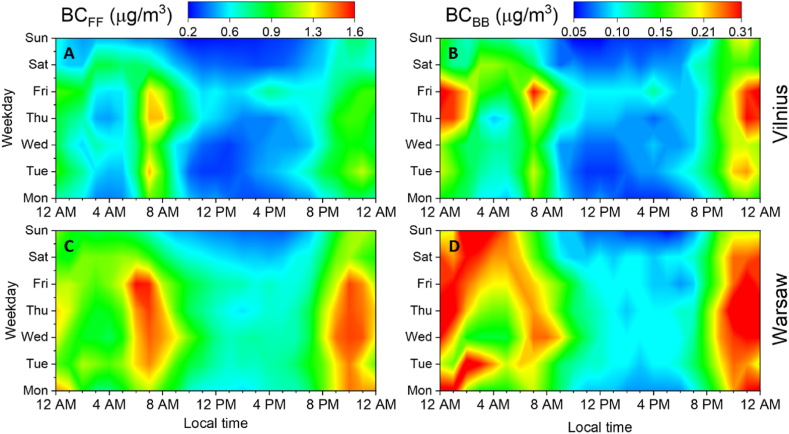
Contour plots of diurnal cycles of BC_FF_ (A and C) and BC_BB_ (B and D) mass concentrations in Vilnius (upper row) and Warsaw (lower row) during the measurement campaign (May–August 2022); note the different colour scale.

The diurnal cycle of BC mass concentration (hourly-averaged, in local time) in Vilnius and Warsaw is shown in Fig. 4. In general, BC_FF_ and BC_BB_ mass concentrations in both cities show similar dynamics during the day. The BC_BB_ mass concentration is 5.2 and 5.9 times lower than the BC_FF_ in Vilnius and Warsaw, respectively. The BC_FF_ follows a consistent pattern throughout the working days (Mon-Fri) with two peaks, in the morning (6–8 a.m.) and late evening (9–11 p.m.) hours (Fig. 4 A, C). The BC_FF_ morning peak in Vilnius (1.04 μg/m^3^) and Warsaw (up to 1.37 μg/m^3^) indicates a strong influence of transport exhaust emissions due to the traffic rush-hours on working days.

Compared to the BC_FF_ mass concentration at night (0–5 a.m.), the morning rush-hour traffic contributes twice higher in Vilnius (56%) than in more polluted Warsaw (28%). Following into the daytime (10 a.m.–5 p.m.), the BC_FF_ mass concentration shows a 50% decrease at both sites due to increasing temperature and mixing layer depth, known to result in pollution dilution [67]. Even though evening rush-hour typically begins around 4–5 p.m. in Vilnius and 5–7 p.m. in Warsaw, the BC_FF_ increase becomes visible only around 6 p.m. and 8 p.m., respectively, when temperature and mixing layer height decrease. The higher pollutant concentrations at night are partly caused by intensifying traffic; changes in mixing layer depth also play a role, which results in pollutant accumulation.

In Vilnius the BC_FF_ diurnal cycle on Fridays (Fig. 4. A) exhibits only a weak peak at 4 p.m. (0.75 μg/m^3^) compared to other working days, which is likely a result of the traffic activity, when most people end their workday earlier and tend to leave the city for the weekend. In Warsaw, the situation is different; the BC_FF_ diurnal cycle on Fridays is similar to other working days. The dynamics of BC_FF_ over the weekend show mass concentrations at both sites being 28% (Vilnius) and 16% (Warsaw) lower than working days. These results indicate that fossil fuel combustion has a much lower impact on weekends. Although there are the same air quality regulations from EU Directive [16] applied in both cities, there still are differences in pollution levels caused by the higher population in Warsaw and people commuting longer distances with cars in a much larger metropolitan area than Vilnius.

The BC_BB_ mass concentration shows different diurnal cycle than BC_FF_ (Fig. 4 B, D). BC_BB_ exhibit variations in distribution between working days and weekdays in both cities. During the weekend, BC_BB_ was 26% and 7% lower in Vilnius and Warsaw, respectively. Given the low mass concentration of BC_BB_ (0.05–0.31 μg/m^3^) and the fact that the demand for burning biomass for domestic heating is relatively low during the studied period (the warm season), BC_BB_ could be considered a background pollutant from leisure activities such as saunas, barbeques, and bonfire burning.

A distinct feature of BC_BB_ in Vilnius is the sharp increase in mass concentration (up to 0.26 μg/m^3^) during Thursday and Friday nights (10 p.m.–2 a.m.) (Fig. 4 B), which can be explained by the large influence of bonfire-burning and barbecuing activities popular in the summer in Lithuania [68]. In Warsaw, BC_BB_ exhibits the highest concentrations at night (9 p.m.–8 a.m.) throughout the week. Higher BC_BB_ mass concentration at night is usually attributed to domestic heating purposes [49,56,69], but this should not be the case in the warm season. Another large BC source could be coal combustion, which is more common in Poland than Lithuania. In the cold season in North-Eastern Poland coal combustion is reported to contribute 41% to the total carbonaceous matter [70]. The aethalometer model apportions BC based on differences in light absorption for biomass burning and fossil fuel combustion but does not take into account coal combustion, which could make a contribution to total BC mass concentration. We initially expected the contribution of coal combustion to be negligible in the warm season. However, the diurnal plots obtained indicate that even during the warm season in Warsaw, coal combustion may have indeed contributed certain amounts to the total BC. If so, this could affect accurate BC apportionment into just two categories. Note that on April 26, 2022, an amended anti-smog resolution for the Mazovia region came into force [71], stipulating that from October 1, 2023, residents of Warsaw are no longer able to burn coal in stoves or fireplaces that are a source of heating, except for residents with class 5 heating boilers. But again, this concerns mainly the cold season (with enhanced heating needs).

### 4.2 Optical characteristics of aerosol particles

We analyzed the SAE, AAE, and SSA to investigate the aerosol particles containing BC during the warm season. Higher mean SAE values were obtained in Vilnius (1.92 ± 0.35) than in Warsaw (1.79 ± 0.39). Knowing that SAE is related to particle size, the results show a larger contribution of submicron particles in Vilnius than in Warsaw. Although SAE is linked to particle size, small differences in SAE values observed between the two sites cannot provide a ground for assessing the specific contribution of submicron particles to the total particle mixture. This highlights the potential for future studies on the relationship between SAE and aerosol size distribution at the two sites.

Monthly mean SAE values followed a similar pattern throughout the measurement period for both sites, increasing in the following order: SAE_May_ < SAE_July_ < SAE_August_ < SAE_June_ (1.52, 1.89, 1.98, 2.09 for Vilnius and 1.60, 1.84, 1.86, 1.91 for Warsaw). The results indicate that submicron particles dominated both sites more in June and August than in other months. The low SAE suggest that supermicron particles could have made the strongest contribution in May at both sites, which could be explained by the high abundance of pollen suspended in the air due to the blooming period of the majority of trees in Lithuania and Poland [72,73].

**Fig. 5:**
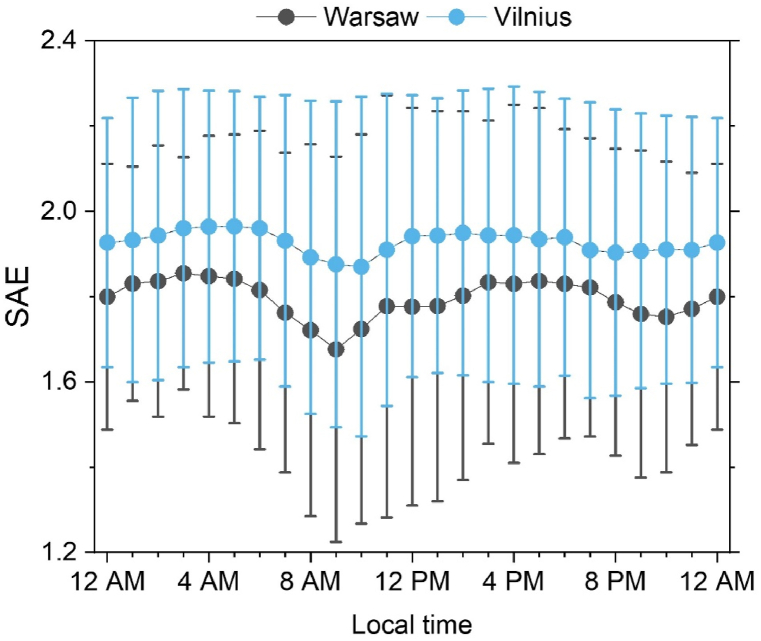
Diurnal cycle of SAE in Vilnius (blue) and Warsaw (grey).

The diurnal analysis of SAE (Fig. 5) reveals the highest values in Vilnius at 5 a.m. (SAE = 1.96 ± 0.33) and Warsaw at 3 a.m. (SAE = 1.85 ± 0.27), thus indicating a significant impact of fine aerosol particles on the aerosol optical properties at night (the higher the SAE, the greater the impact of smaller particles). The lowest SAE values were recorded at 10 a.m. in Vilnius (SAE = 1.88 ± 0.38) and 9 a.m. in Warsaw (SAE = 1.68 ± 0.45). The decreasing SAE values during the hours demonstrate the impact of particle growth, which occurs due to the intensification of aerosol particle sources (exhaust and non-exhaust traffic emissions, organic aerosol formation) [[74], [75], [76]].

**Fig. 6:**
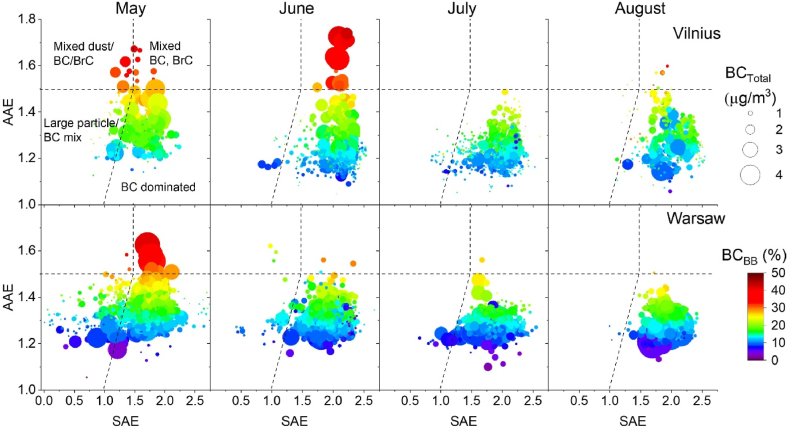
Relationship between AAE, SAE, total BC_Total_ mass concentration, and biomass burning (BC_BB_) contribution in May–August 2022 in Vilnius (upper row) and Warsaw (lower row). Classification scheme proposed by Cappa et al. [62].

The plots of SAE vs. AAE, BC_Total_, and BC_BB_ shown in Fig. 6 indicate the source contribution and chemical composition of aerosol particles. In general, during the measurement campaign, values fell into four regions or scenarios, as follows: “BC dominated” (most highly abundant), “Large particle/BC mix”, “Mixed BC, BrC”, and “Mixed dust/BC/BrC” (least abundant). Indeed, for all months from May to August 2022, majority of points (90%) at Vilnius and Warsaw were distributed in the “BC dominated” region. Specifically interesting is that months of July, August in Vilnius and June, July, August in Warsaw are highly similar regardless location and type of the two sites. In Warsaw, the “Large particle/BC mix” region received a number of points in May (25%) as well as “Mixed BC, BrC” (3%). Thus, the aerosol particles in May significantly differed from the remaining months (from June to August). In Vilnius, some points were distributed in the region “Mixed dust/BC/BrC” (1% in May) and “Mixed BC, BrC (1.5% in June).

During the entire measurement campaign, the highest BC mass concentration (up to 4.4 μg/m^3^) and highest biomass burning contribution to BC (43%) are observed in the region “Mixed BC, BrC” in June in Vilnius. These points correspond to June 24, 2022, which is Midsummer, a Lithuanian national holiday vibrantly celebrated with bonfires and other outdoor festivities. As a result, this significant contribution of biomass burning can be attributed to leisure activities, not domestic heating. Due to cultural differences, no such observations were recorded in Warsaw. Notably, a similar feature with points in the region “Mixed BC, BrC” with the highest BC mass concentration (up to 4 μg/m^3^) and highest biomass burning contribution to BC (42%), can be seen in May in Warsaw. This can be attributed (similar to the aforementioned case of Midsummer celebrations) to the May Days celebrations on 1st–3rd May 2022, starting on 1st May (Labour Day), 2nd May (Flag Day), and 3rd May (Constitution Day). During the abovementioned days, there were several facilities whose regulations exceptionally allowed the possibility of lighting a barbeque (for mobile and stationary grills) in strictly selected areas; one of these areas was in the municipal park Pole Mokotowskie, just a few hundred metres from the measurement site.

Comparably similar AAE vs. SAE plots were obtained for June, July, and August in Warsaw as well as July and August in Vilnius, when the points almost entirely allocated to the “BC dominated” region. The low abundance of high BC_BB_ contributions to BC_Total_ values and the near absence of data points in the AAE >1.5 region could indicate a decrease in biomass burning for domestic heating, which raises the concentration of BC_BB_ and BrC. Although during summer, plumes of aerosol particles from bonfires can be observed at different locations [68,77], the sites in our study (urban or urban background) seem not to reflect such activities, which are anyhow more popular outside the city, except for the two cases discussed above (Midsummer in Vilnius and May Days in Warsaw).

The SSA as a ratio of scattering versus the sum of scattering and absorption coefficients, depends on the size of a given particle, its absorbing properties, and hence its origin. Aerosol particles containing light-scattering inorganics (e.g., sulphate, nitrate, and sea salt) have higher SSA values (up to 1), whereas light-absorbing aerosol components shift SSA to lower values [78]. In our study, mean SSA values (not shown for brevity) for the 2022 warm season in Vilnius and Warsaw were very low: 0.72 (±0.1) and 0.69 (±0.1), respectively. Monthly averaged SSA values for May, June, July, and August in Vilnius were 0.69 (±0.08), 0.73 (±0.1), 0.71 (±0.1), and 0.72 (±0.11) and in Warsaw 0.66 (±0.1), 0.72 (±0.09), 0.68 (±0.1), and 0.70 (±0.11), respectively. In Vilnius, the range of SSA was wider (from 0.17 to 0.98) than in Warsaw (0.3–0.9). For both measurement sites, the mass concentration of BC_FF_ showed a low negative correlation with SSA values: 0.48 in Vilnius and −0.46 in Warsaw. Given that higher BC_FF_ concentrations resulted in lower SSA values, it is confirmed that fossil fuel combustion in Warsaw and Vilnius had a greater impact on warming the atmosphere than biomass burning, bearing in mind that the obtained correlation values are not statistically significant.

### 4.3 Role of long-range air mass transport

As a final step, we investigated to what extent the long-range transport of aerosol particles may play a role in altering its properties. We focused on the BC mass concentration and its link to the calculated HYSPLIT backward trajectories. The trajectories (in total 112) were separated into categories A, B, C. In category A, the overlapping trajectories from similar direction and uniform in H- and/or V-thresholds were found for a large number of 47 cases. Note that they are split further in terms of the time parameter to Aa: long uniform trajectories overlapping for at least 36 h found in 7 cases; Ab: short uniform trajectories overlapping for less than 12 h found in 25 cases; and Ac: semi uniform trajectories parallel only in H-threshold, separately for 500 m and 1000 m found in 15 cases. In category B, the strictly different source and pathway trajectories in horizontal and vertical directions were found for 17 cases. In category C, the non-classified trajectories remained for 48 cases. Analysis shows that the number of cases found with strictly non-overlapping trajectories (B) was almost three times lower than for overlapping trajectories (A), thus we see the mesoscale meteorology influence at both sites. In the latter category (A), the long uniform trajectories (Aa) are seldom (15%), the semi-uniform trajectories (Ac) occur twice as often (30%), while the most often the short-uniform trajectories (Ab) appear (53%).

**Fig. 7:**
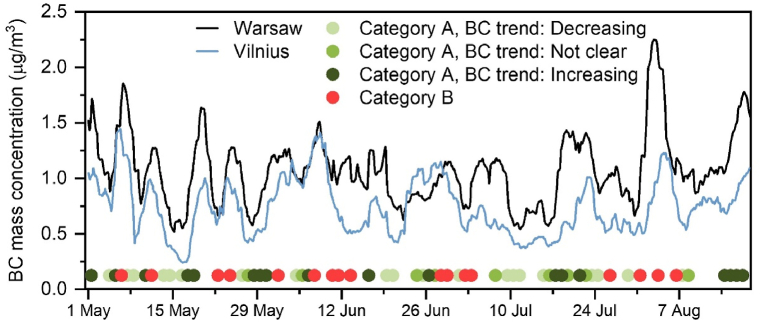
Time series of 72-hr running mean of BC mass concentration in Vilnius (blue) and Warsaw (black) in May–August 2022. The circles mark air mass trajectory classification: category A – overlapping trajectories (47 cases), category B – different source and pathway (17 cases). Based on the BC behaviour for both sites, the overlapping cases are further classified into light green (clear simultaneous decrease) and dark green (clear simultaneous increase).

For category A, the BC behaviour assessed by discriminating the number of cases of pollution boost vs dilution in consecutive 6-hr intervals, the decrease of BC values simultaneously at both sites was found for 20 cases and the increase for 18 cases. There were only 9 cases with no clear or opposite trend found. This indicates that air masses transported along the overlapping trajectories and arriving at the same time at both sites must significantly affect the local air pollution. In Fig. 7, we show the time series of the 72-hr running mean of BC mass concentration in Vilnius and Warsaw from May to August 2022. The circles denote classification results: transport of air masses assessed with respect to uniform air mass flow, with overlapping backward trajectories arriving at both sites was observed in 42% of the data (category A, green dots). Distinctly non-uniform flows were found for only 16% of the data (category B, red dots), with air masses following strictly differing trajectories. The cases of uniform air mass transport correlate with a simultaneous increase or decrease of BC concentrations at both sites, as depicted in Fig. 7, clearly indicating a decreasing (20 cases in light green dots) or increasing (18 cases in dark green dots) behaviour. For the overlapping trajectories that have similar long-range transport sources and pathways, a simultaneous decrease in BC at both sites can be explained as a sort of ventilation process diluting pollution occurring at both locations (e.g., due to clean air masses moving in from Northern Europe). On the contrary, a simultaneous increase may result from a boost in local pollution at both sites (e.g., transporting polluted air from Western or Eastern Europe). It is known that long-range air mass transport can affect the aerosol properties within the boundary layer over long distances (e.g., anthropogenic pollution intrusion from Germany to Warsaw [79], or biomass burning intrusions from Ukraine [47]). Such intrusions can cause opposite correlations of the Ångström Exponent vs. the boundary layer height and the aerosol optical depth, depending on the type of the intruding aerosol.

**Fig. 8:**
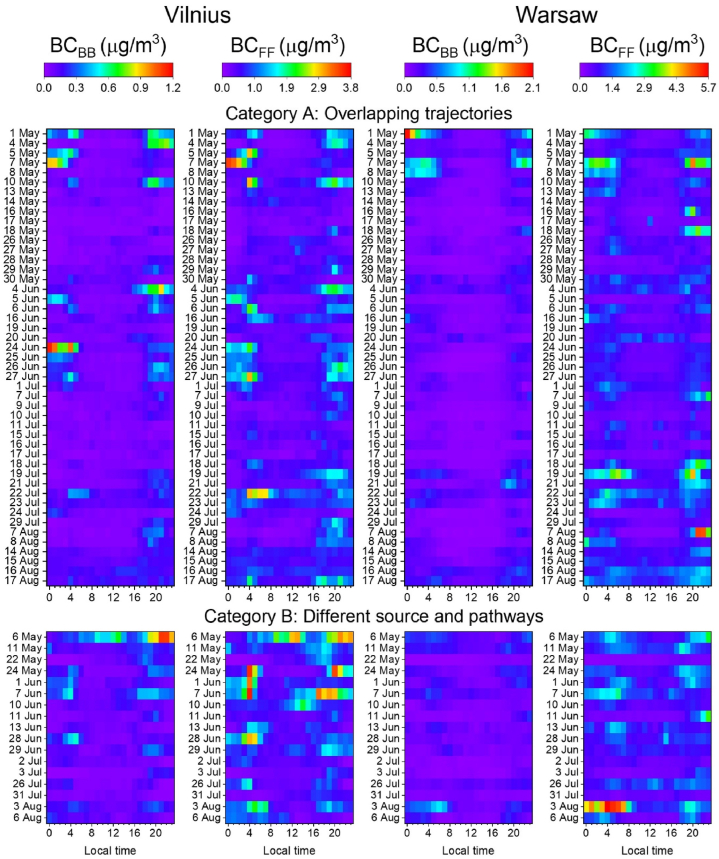
Heat maps of BC_BB_ and BC_FF_ mass concentration diurnal cycles in Vilnius and Warsaw during the days when 72-hr backward trajectories were apportioned to category A (upper row) and category B (lower row); note the different colour scale.

To assess if there are patterns of similarities in the obtained diurnal cycles at each site with respect to BC_FF_ and BC_BB_ apportionment, we depicted the obtained results in the consecutive rows of Fig. 8. As it must be expected, there are no patterns of similarities for the data classified in the category B. This is due to differing sources of air masses and the inconsistent directions of air-mass pathways, which did not have a chance to “encounter” each other. If there would be any similarities found, they must have been accidental. However, for category A one can see that there are more patterns of similarities, both due to the ventilation process (decreasing BC) or boosts in pollution (increasing BC). In general, the effect of the BC decrease was similar at both sites (42% in Warsaw, and 50% in Vilnius) but the effect of increase was more significant for Vilnius (64%) than for Warsaw (30%). The pollution boosts are more significant for BC_FF_ (increase of 27% in Warsaw and 50% in Vilnius) than for BC_BB_ (similar increase of 7% in Warsaw and 11% in Vilnius). The BC_FF_ pollution dilution was less significant in Warsaw (decrease of 34%) than in Vilnius (51%) and the BC_BB_ dilution was comparable (8% in Warsaw and 5% in Vilnius). This indicates that biomass burning-related pollution from long-range transport was not as important as local sources of this pollution type (e.g., very high pollution on 1st May in Warsaw due to May Days and 24th June in Vilnius due to Midsummer celebrations; see Fig. 8 category A). In contrast, pollution due to fossil fuel combustion is affected by long-range transport and results in equally important pollution dilution and boosts.

## 5 Conclusions

The aethalometer and nephelometer measurements during the warm season of 2022 (May–August) revealed a 29% lower BC mass concentration at the urban background site in Vilnius (Lithuania) (0.77 μg/m^3^) than at the urban site in Warsaw (Poland) (1.07 μg/m^3^). Despite the contrasting nature of the sites, BC source apportionment to biomass burning and fossil fuel combustion showed similar contributions: 16% and 84% in Vilnius and 14% and 86% in Warsaw for BC_BB_ and BC_FF_, respectively. The weekly patterns of BC were dominated by BC_FF_ contribution, with lower concentrations on the weekends. The diurnal BC cycle (dominated by fossil fuel combustion) showed clear morning and evening traffic peaks at both cities. Separation of aerosol components based on AAE and SAE revealed that submicron BC-dominated particles were the most abundant at both sites (90%) for every month. The two local episodes of biomass burning during Midsummer in Vilnius (24th June) and May Days in Warsaw (1st – 3rd May) were distinctly different in terms of AAE (1.5<AAE<1.7), with a higher mixture of BC and BrC. SSA values observed in Warsaw, with a mean value of 0.69 (±0.1), and in Vilnius, with a mean value of 0.72 (±0.1), exhibited a clear negative correlation with BC_FF_, with corresponding correlation coefficients of −0.46 and −0.48, respectively. This strongly supports the conclusion that fossil fuel combustion significantly influences the radiative properties of aerosol particles in both cities.

The long-range air mass transport effect on the BC mass concentration in terms of simultaneous pollution boost or dilution at the two sites was performed using the proposed threshold-based classification of HYSPLIT backward trajectories. The long-range air masses arriving uniformly from similar directions had a similar effect on the BC decrease (42% in Warsaw, 50% in Vilnius). Although the effect of BC increase was twice stronger in Vilnius (64%) compared to Warsaw (30%). The pollution boost and dilution were more significant for BC_FF_ in Vilnius (boosts of 50%, dilution of 51%) compared to Warsaw (boosts of 27%, dilution of 34%). While the BC_BB_ effect was comparable for both cities (boosts of 11%, dilution of 5% in Vilnius and boosts of 7%, dilution of 8% in Warsaw).

Overall, our findings show that, during the warm season, biomass burning contributes significantly less to atmospheric composition in Warsaw and Vilnius than fossil fuel combustion, except for the local holiday-related episodes.

## Funding

The joint Lithuanian-Polish research project DAINA-2 grant “Importance of long-range transport of BIOmass burning emissions to local Smog events in Urban Environments (BIOSURE)” is supported by the National Science Centre of Poland (Narodowe Centrum Nauki; Grant No. 2020/38/L/ST10/00480) and the 10.13039/501100004504Research Council of Lithuania (Lietuvos mokslo taryba; Grant No. S-LL-21-7). Regular measurements at the Warsaw site are supported long-term by 10.13039/501100000780EC 10.13039/501100007601Horizon 2020, GAs No. 871115 (ACTRIS-10.13039/501100009893IMP).

## Data availability

The data used in this study are openly available at the ICM-UW RepOD Data Repository under Minderytė, Agnė; Byčenkienė, Steigvilė; Stachlewska, Iwona, 2023, "Black carbon properties for warm season May**–**August 2022 at two urban sites in Vilnius (Lithuania) and Warsaw (Poland)", https://doi.org/10.18150/BCUBJK, RepOD, V1.

## Author contribution

Agnė Minderytė: Analyzed and interpreted the data; Wrote the paper.

Emeka A. Ugboma: Analyzed and interpreted the data; Wrote the paper.

Fátima Francisca Mirza Montoro: Analyzed and interpreted the data.

Iwona S. Stachlewska: Conceived and designed the experiments; Performed the experiments; Contributed reagents, materials, analysis tools or data; Wrote the paper.

Steigvilė Byčenkienė: Conceived and designed the experiments; Contributed reagents, materials, analysis tools or data.

## Additional information

No additional information is available for this paper.

## Declaration of competing interest

The authors declare that they have no known competing financial interests or personal relationships that could have appeared to influence the work reported in this paper.
